# A Case of Disseminated Superficial Actinic Porokeratosis Successfully Treated With Topical Lovastatin/Cholesterol Gel

**DOI:** 10.7759/cureus.40582

**Published:** 2023-06-17

**Authors:** Qiret Sultan, Blaine Massey, David G Cotter

**Affiliations:** 1 Department of Internal Medicine, Kirk Kerkorian School of Medicine at University of Nevada, Las Vegas, Las Vegas, USA; 2 Department of Dermatology, Las Vegas Dermatology, Las Vegas, USA

**Keywords:** topical lovastatin, topical cholesterol, mevalonate, porokeratosis, dsap, disseminated superficial actinic porokeratosis

## Abstract

Disseminated superficial actinic porokeratosis (DSAP) is a disorder of abnormal keratinization for which there is no standard treatment. Treatment modalities that have traditionally been utilized with varying success include ablative therapies, topical pharmacologic treatments, surgical excision, and retinoids. The underlying pathophysiology of DSAP is secondary to genetic mutations in the mevalonate biosynthesis pathway, and thus topical lovastatin/cholesterol presents a promising treatment modality for this condition. We present a case of familial DSAP successfully treated with topical lovastatin/cholesterol gel and provide a brief review of the existing literature surrounding this novel therapy.

## Introduction

Disseminated superficial actinic porokeratosis (DSAP) is a condition characterized by clonal proliferation of atypical keratinocytes that form erythematous, pruritic cutaneous lesions surrounded by a thin, threadlike keratotic border. While sporadic cases may arise, familial DSAP typically demonstrates an autosomal dominant pattern of inheritance with incomplete penetrance due to loss-of-function mutations in the mevalonate biosynthesis pathway that result in cholesterol deficiency with subsequent impaired skin barrier function [1,2]. Thus, modulation of cholesterol metabolism presents an intriguing therapeutic target.

The variants of porokeratosis include DSAP, disseminated superficial porokeratosis, classic porokeratosis (CP) of Mibelli, linear porokeratosis, and porokeratosis palmaris et plantaris disseminata (PPPD). CP usually appears at an early age on the skin and mucous membranes. PPPD appears in young adulthood with lesions on the palms and soles [3]. Linear porokeratosis arises in childhood, presenting as unilateral, linear, hyperkeratotic papules and plaques with associated nail dystrophy in some cases [4]. Compared to other forms of porokeratosis, familial DSAP is notable for its characteristic photodistribution, onset in the third to fourth decade, a large number of lesions, and family history [1]. 

DSAP can be diagnosed by clinical exam and dermoscopy. Characteristic dermoscopy findings include a peripheral keratotic rim, central atrophy with a scar-like appearance, and linear and pinpoint vessels [5]. Biopsy is not necessary for diagnosis but can be performed if the diagnosis is uncertain. The histology of DSAP lesions demonstrates a characteristic cornoid lamella, which represents a column of parakeratotic cells overlying a thin granular layer with edematous keratinocytes and scattered lymphocytes [1]. DSAP is historically difficult to treat.

## Case presentation

**Figure 1:**
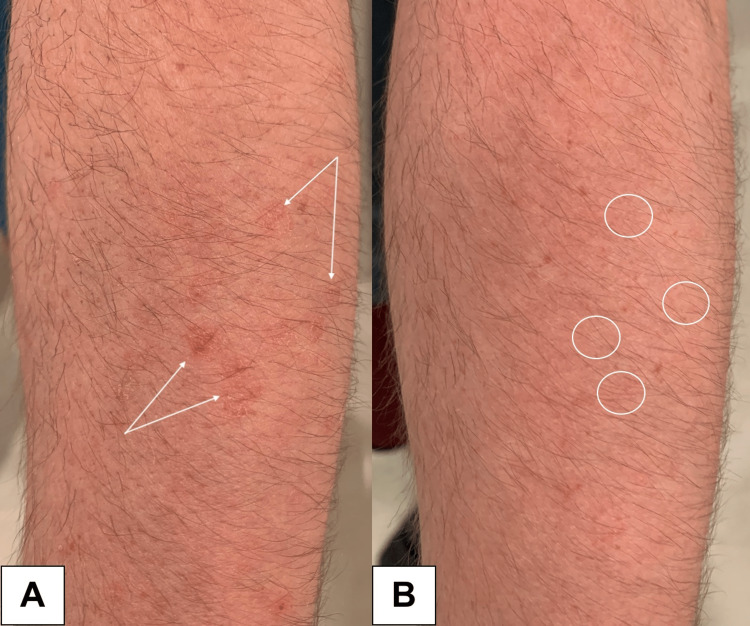
A. White arrows indicate DSAP lesions on the left forearm before treatment. B. White circles highlight areas of lesion resolution on the left forearm after two months of treatment. DSAP: disseminated superficial actinic porokeratosis.

**Figure 2:**
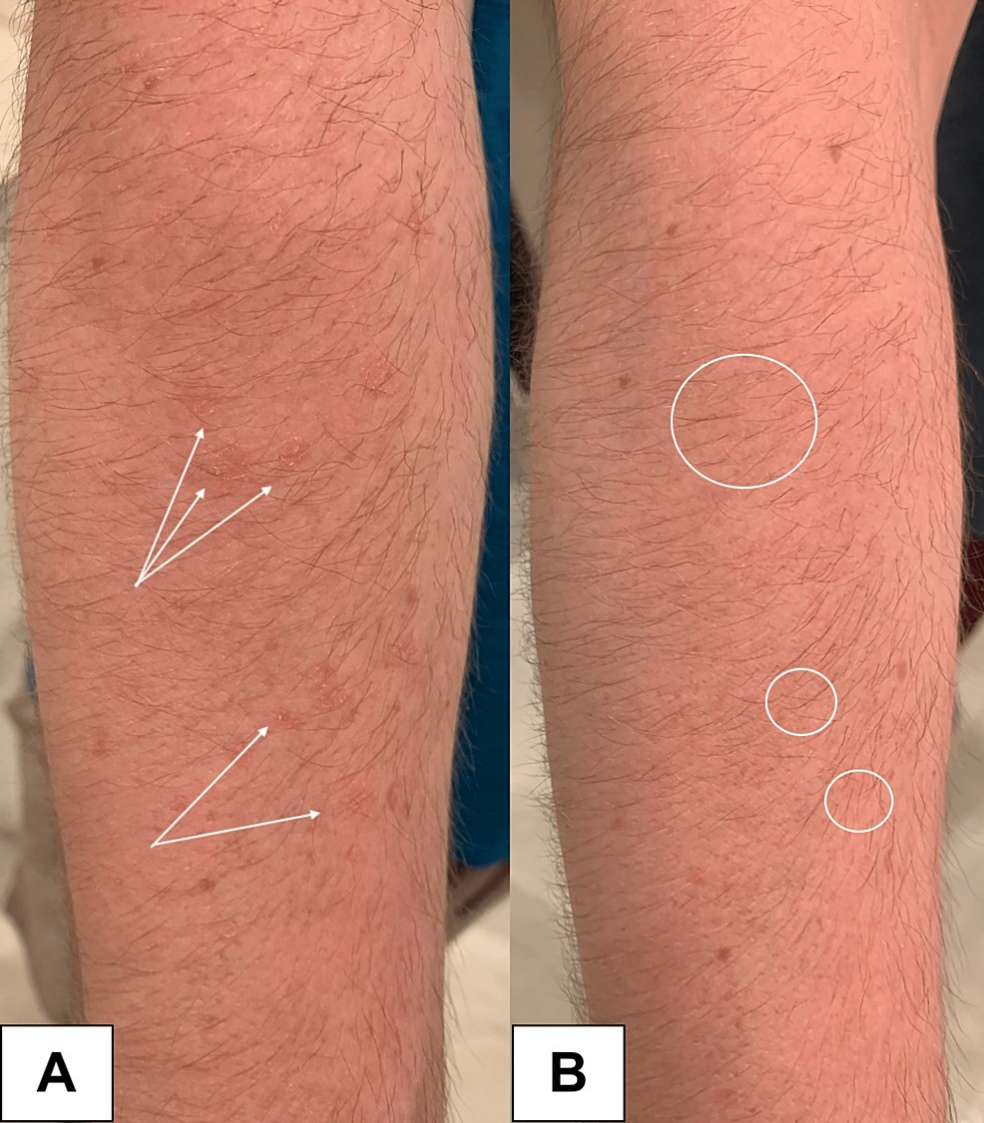
A. White arrows indicate DSAP lesions on the right forearm before treatment. B. White circles highlight areas of lesion resolution on the right forearm after two months of treatment. DSAP: disseminated superficial actinic porokeratosis.

A 30-year-old man presented to the dermatology clinic with pinkish-orange, thin, round papules with a threadlike border on the extensor forearms (Figures 1A, 2A). 

The lesions arose in his early twenties, exhibited photo-exacerbation, and were asymptomatic, aside from cosmetic disfigurement. Family history was significant for DSAP in his half-brother. Dermoscopy revealed annular red-pink papules with central atrophy and a peripheral keratotic rim (cornoid lamella).

A clinical diagnosis of familial DSAP was made. No biopsy or genetic testing was performed. The patient had previously failed various treatment modalities including pulsed-dye laser (PDL), erbium-doped yttrium aluminum garnet (Er:YAG) resurfacing, and topical tretinoin. 

Field treatment with compounded topical 2% lovastatin/cholesterol gel (CareFirst Specialty Pharmacy, Mount Laurel, New Jersey) once daily was initiated. At one month follow-up, the lesions demonstrated marked diminution in size, erythema, and scaling, but remained visible on close inspection. After two months of topical treatment, the lesions exhibited complete remission (Figures 1B, 2B). 

The patient experienced no adverse events or reactions. Once the lesions were in remission, the patient decided to switch to every other day application, which has been adequate for maintenance. However, he does occasionally note lesion flares upon increased ultraviolet (UV) light exposure, and returns to daily treatment when this occurs.

## Discussion

**Figure 3:**
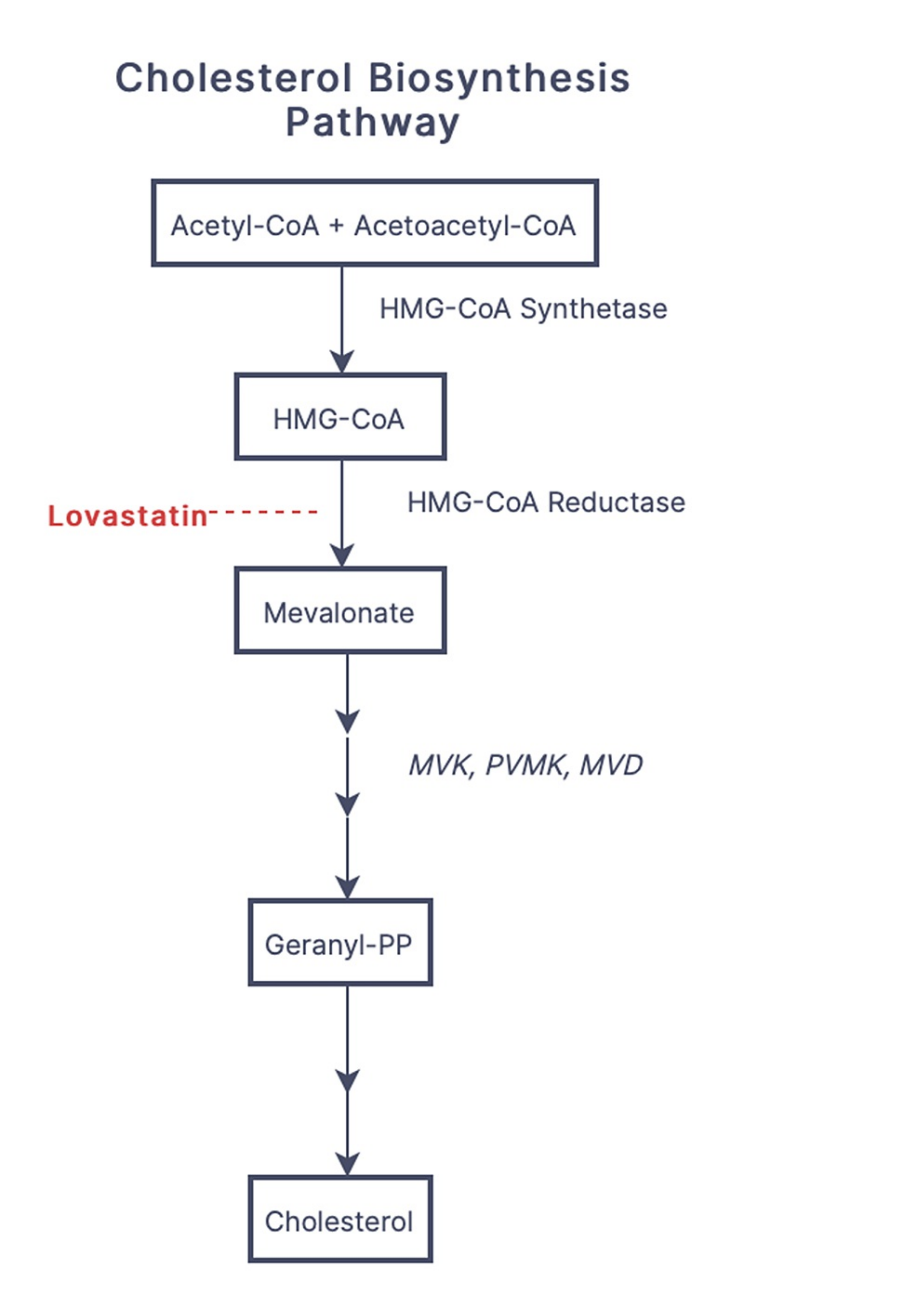
Flowchart depicting cholesterol biosynthesis pathway. The site of action of lovastatin is depicted in red. Genes implicated in DSAP pathogenesis are italicized. Acetyl-CoA: acetyl-coenzyme A; acetoacetyl-CoA: acetoacetyl-coenzyme A; HMG-CoA: β-hydroxy β-methylglutaryl-coenzyme A; MVK: mevalonate kinase; PVMK: phosphomevalonate kinase; MVD: mevalonate diphosphate decarboxylase; Geranyl-PP: geranyl pyrophosphate; DSAP: disseminated superficial actinic porokeratosis.

DSAP is the most common of the porokeratosis variants. However, the exact prevalence is unknown. The underlying pathophysiology of DSAP is due to loss-of-function mutations in mevalonate kinase (MVK), phosphomevalonate kinase (PMVK), and mevalonate diphosphate decarboxylase (MVD) genes of the cholesterol biosynthesis pathway (Figure 3), resulting in accumulation of cytotoxic metabolites and impaired protection against UV light.

Before the diagnosis of DSAP, the patient was seen by a different provider and treated with PDL, Er:YAG laser, and topical tretinoin. The patient reported no response to PDL or topical tretinoin. He reported temporary resolution of lesions with Er:YAG ablation; however, the lesions recurred within several months. When he presented to our dermatology clinic, a diagnosis of DSAP was made. The decision to try topical lovastatin/cholesterol was based on novel literature supporting this modality.

The use of statins may be useful in the treatment of dermatological conditions due to their anti-inflammatory effects. Specifically, topical statins have been shown to have therapeutic benefits in the treatment of acute irritant contact dermatitis, acne, rosacea, and rhinophyma [6]. The proposed mechanism of topical lovastatin involves the inhibition of cytotoxic metabolites formed within the mevalonate pathway. Cholesterol is added to replenish the end product of this pathway to maintain cell integrity and stabilize the extracellular lipid matrix. Insufficient levels of cholesterol appear to increase keratinocyte susceptibility to apoptosis and disrupt cellular differentiation. The statin component appears to be the key therapeutic agent, as topical cholesterol alone has not shown clinical benefit [7,8]. 

While no randomized controlled trials have been performed to evaluate the treatment of DSAP, small-scale studies have investigated the utility of various treatment options. Treatments previously utilized for DSAP include topical diclofenac, corticosteroids, 5-fluorouracil, retinoids, imiquimod, cryotherapy, and laser ablation therapy with inconsistent results [1,9]. A literature search of DSAP treatment with topical statin/cholesterol yielded one open-label split body clinical trial, two case series, and one case report with a total of 21 patients evaluated [7-10]. All patients (n=21) demonstrated objective improvement and/or complete resolution of lesions. Mild adverse effects were reported in 19% (n=4) of patients, with temporary irritation being the most common. Treatment with topical lovastatin/cholesterol vs lovastatin alone for the treatment of DSAP is currently under investigation (Clinicaltrials.gov, NCT0435982) [11]. 

Primary care physicians should be aware that DSAP may mimic actinic keratosis, seborrheic keratosis, psoriasis, lichen planus, and more [12,13]. DSAP lesions may carry a small risk of malignant transformation, up to 6.9%-11.6%, with squamous cell carcinoma being the most common, followed by basal cell carcinoma and melanoma [14]. Some studies suggest that DSAP may have a higher risk of malignant transformation compared to other porokeratosis variants; therefore, accurate and timely identification of DSAP lesions with appropriate treatment is crucial in preventing future morbidity and mortality [15]. As DSAP lesions offer a low risk of malignant transformation, annual surveillance by a dermatologist is prudent. Treatment targeting the underlying pathophysiology of this condition appears to be safe and effective. Sun protection or avoidance should also be emphasized to the patient. This case supports continued investigation of topical lovastatin/cholesterol to treat DSAP.

## Conclusions

Many different treatment options have been used for the management of DSAP with varying results. Modulation of the cholesterol biosynthesis pathway through treatment with topical lovastatin/cholesterol gel directly targets the toxic metabolites implicated in DSAP pathogenesis. This treatment appears to induce remission and markedly reduce disease activity in DSAP lesions. Clinicians may consider topical lovastatin/cholesterol gel in the treatment of DSAP due to its accessibility, cost-effectiveness, and efficacy compared to other treatments. Adequately powered prospective randomized trials are needed to further evaluate the safety and efficacy of this treatment.
